# With Great Power Comes Great Responsibility: Common Errors in Meta-Analyses and Meta-Regressions in Strength & Conditioning Research

**DOI:** 10.1007/s40279-022-01766-0

**Published:** 2022-10-08

**Authors:** Daniel Kadlec, Kristin L. Sainani, Sophia Nimphius

**Affiliations:** 1 School of Medical and Health Sciences, Centre for Human Performance, Edith Cowan University, 270 Joondalup Drive, Joondalup, WA 6027 Australia; 2 Epidemiology and Population Health, Stanford University, Stanford, CA USA

## Abstract

**Background and Objective:**

Meta-analysis and meta-regression are often highly cited and may influence practice. Unfortunately, statistical errors in meta-analyses are widespread and can lead to flawed conclusions. The purpose of this article was to review common statistical errors in meta-analyses and to document their frequency in highly cited meta-analyses from strength and conditioning research.

**Methods:**

We identified five errors in one highly cited meta-regression from strength and conditioning research: implausible outliers; overestimated effect sizes that arise from confusing standard deviation with standard error; failure to account for correlated observations; failure to account for within-study variance; and a focus on within-group rather than between-group results. We then quantified the frequency of these errors in 20 of the most highly cited meta-analyses in the field of strength and conditioning research from the past 20 years.

**Results:**

We found that 85% of the 20 most highly cited meta-analyses in strength and conditioning research contained statistical errors. Almost half (45%) contained at least one effect size that was mistakenly calculated using standard error rather than standard deviation. In several cases, this resulted in obviously wrong effect sizes, for example, effect sizes of 11 or 14 standard deviations. Additionally, 45% failed to account for correlated observations despite including numerous effect sizes from the same study and often from the same group within the same study.

**Conclusions:**

Statistical errors in meta-analysis and meta-regression are common in strength and conditioning research. We highlight five errors that authors, editors, and readers should check for when preparing or critically reviewing meta-analyses.

**Supplementary Information:**

The online version contains supplementary material available at 10.1007/s40279-022-01766-0.

## Key Points

**Table:** 

A meta-analysis combines data from single studies to test specific hypotheses, but statistical errors can substantially impact the calculated results and lead to flawed conclusions.
We describe five common statistical errors that are easy to spot and serious enough to markedly impact results.
We identified statistical errors in 85% of the 20 most highly cited meta-analyses in strength and conditioning research over the past 20 years.
Sixty percent of all effect sizes (standardized mean differences) greater than 3.0 were due to a standard error/standard deviation mix-up, meaning that effect sizes this large should have a high index of suspicion for error.
Understanding common sources of statistical error in meta-analyses helps the reader evaluate published research.

## Introduction

Meta-analysis and meta-regression combine data from single studies to test specific hypotheses. Because they provide more robust evidence than single studies, they are often highly cited and may directly influence clinical practice. However, statistical errors in meta-analysis/meta-regression are widespread and can lead to flawed conclusions [1–3]. In this article, we highlight five common statistical errors that we believe are both (1) easy to detect and (2) serious enough to markedly impact results. We first illustrate these errors and their impact using a specific example meta-analysis from strength and conditioning research. We chose this example simply because it came to our attention first. We then attempt to quantify the frequency of these errors by systematically reviewing 20 highly cited meta-analyses from strength and conditioning. Finally, we present a checklist to help authors, reviewers, and editors flag these errors.

## Part 1: Illustrative Example

Seitz et al. [4] extracted data from 15 studies [5–19] that measured both lower-body muscle strength using a free-weight (full, parallel, or half) back-squat exercise and sprint performance before and after a lower-body resistance-training intervention. They reported a large and significant correlation (*r* =  − 0.77 [− 0.85 to − 0.67], *p* ≤ 0.001) between improvements in lower-body muscle strength and improvements in sprint performance and concluded that increases in lower-body muscle strength positively transfer to sprint performance. As of August 2022, Seitz et al. [4] has been cited 147 (Scopus) and 284 (Google Scholar) times. However, a closer inspection of the study reveals important errors, which we describe below. As we will show in Part 2, the identified errors are common in the published literature and Seitz et al. [4] serves merely as an example.

### Ignoring Outliers

An outlier is an extreme case that seems to be well separated from the rest of the data. There is no single way of identifying outliers and they are dependent on the context; however, some commonly used rules of thumb are values that are more than 3 standard deviations from the mean or more than 1.5 times the interquartile range from the median. An outlier in a meta-analysis or meta-regression can affect the validity and robustness of the conclusion [20].

**Fig. 1:**
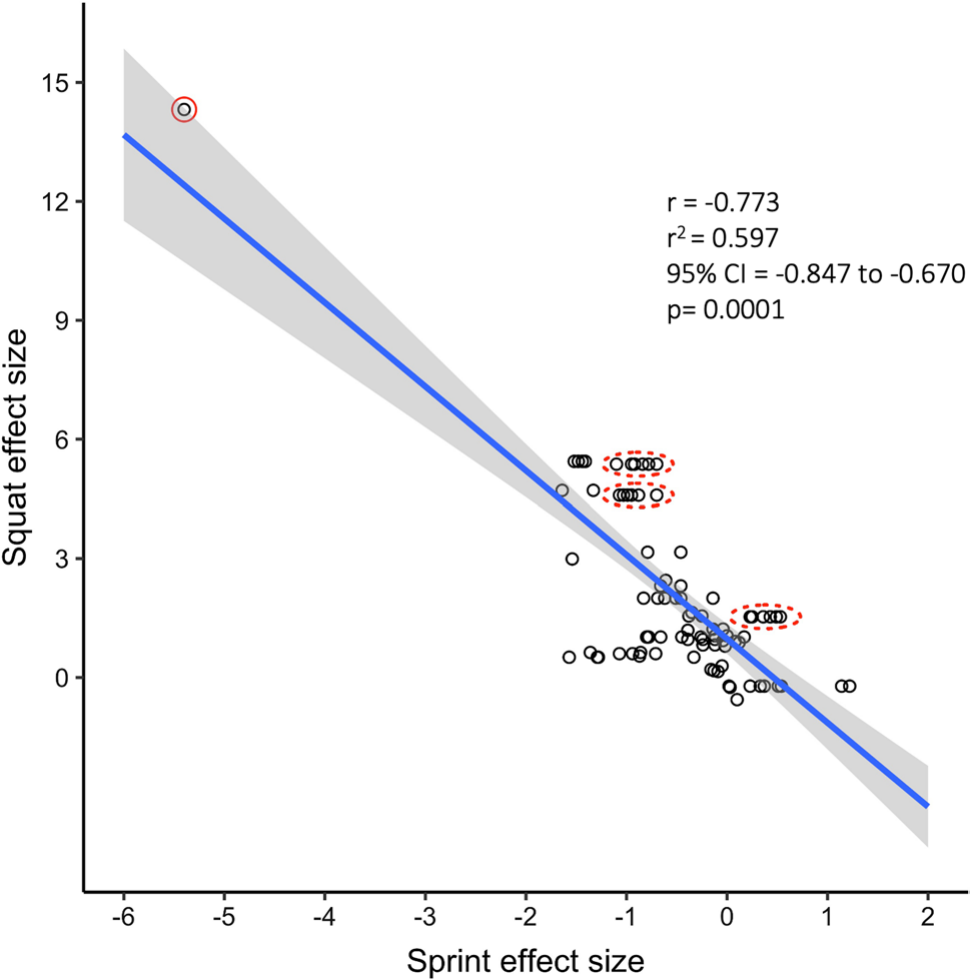
Reproduced figure from Seitz et al. [4] (Fig. 2 of Seitz et al. [4]). *Red circles* have been added to highlight specific statistical issues. The figure shows a scatter plot of squat and sprint Hedges’ g effect sizes (*n* = 85 effect sizes from 15 studies). The blue line is the linear regression line, and the gray cloud shows the 95% confidence interval (CI). The solid red circle highlights an outlier and the dashed red circles highlight examples of highly correlated observations — these stripes of data arise because the same group was subjected to multiple related sprint measurements. *r* coefficient, *r*^*2*^ shared variance

Figure 2 in Seitz et al. [4] displays a conspicuous outlier (reproduced here as Fig. 1, the outlier is circled in red). The graph displays Hedges’ *g* effect sizes, reflecting standardized within-group improvements in sprint performance (decrease in time) and within-group improvements in squat strength [21]. The datapoint derived from Wong et al. [19] indicates an improvement in sprint performance of over 5 standard deviations and an improvement in the squat performance of over 14 standard deviations. Common sense tells us that such improvements are implausibly large. Indeed, in the study that underlies the datapoint [19], a 14-standard deviation improvement in squatting would be about a 110-kg improvement *on average* over an 8-week training period, which is highly implausible from a biological standpoint. The datapoint is erroneous, as we will explain below.

**Fig. 2:**
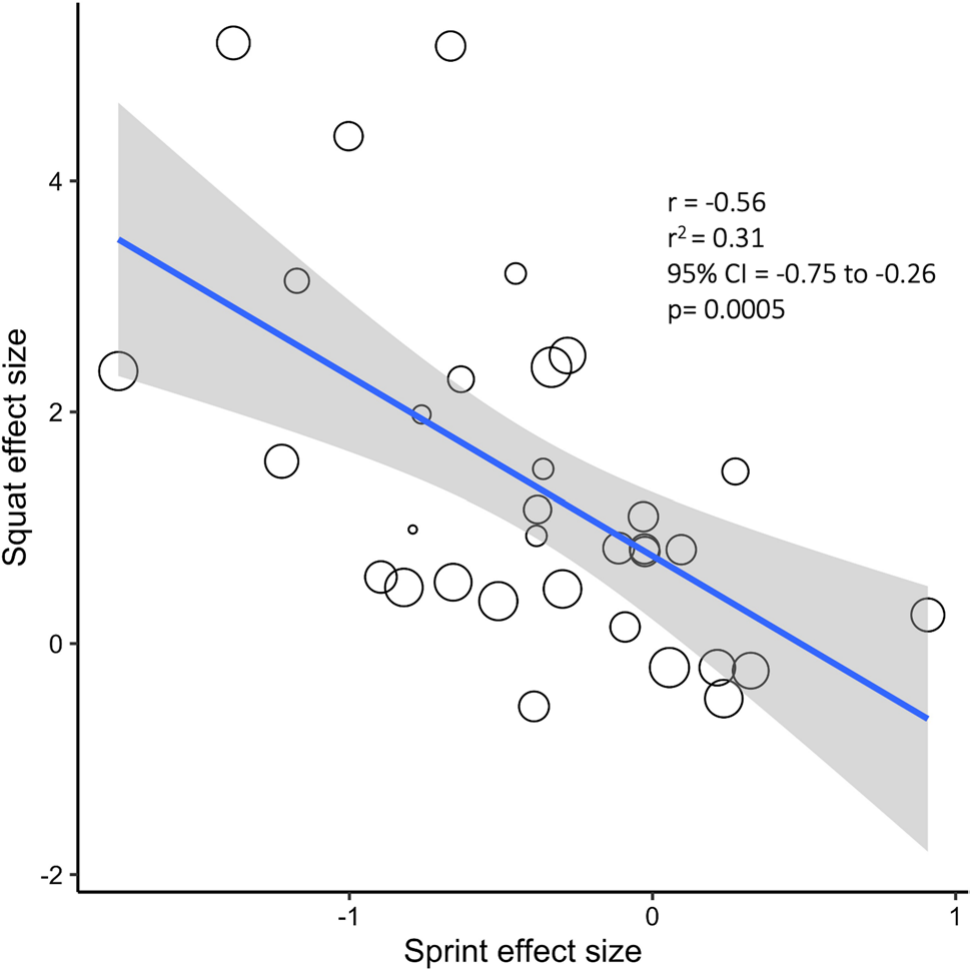
Recalculated correlation between squat and sprint effect sizes (*n* = 33). The size of the datapoint indicates the weighting of the study. The blue line is the linear regression line, and the gray cloud shows the 95% confidence interval (CI). *r* coefficient, *r*^*2*^ shared variance

After spotting an outlier, researchers should first check to make sure the datapoint is real and not an error. Obviously, if the datapoint is erroneous — as is the case in Seitz et al. [4]—the error should be corrected. If found to be real, then researchers should analyze the data with and without the outlier to gauge the influence on the results. The influence of individual studies on a meta-analysis result is examinable by many different approaches (e.g., Baujat plot and influence diagnostics) using freely available R packages {dmetar} [22]. Failure to take these steps can lead to misleading results. In Seitz et al. [4] (Fig. 1), the outlier artificially inflates the estimated correlation coefficient.

### Miscalculated Effect Sizes That Arise From Using Standard Errors Instead of Standard Deviations

Meta-analyses often use effect size measures that incorporate the standard deviation of the outcome measure [23]. However, meta-analysts sometimes confuse standard error and standard deviation and mistakenly extract the standard error rather than the standard deviation from the underlying papers. Using the standard error rather than standard deviation when calculating standardized effect sizes will artificially inflate the values. For example, the equation for Hedges’ g is:

$$g = \frac{{{\text{Mean}}_{{{\text{post}}}} - {\text{Mean}}_{{{\text{pre}}}} }}{{\text{Pooled Standard Deviation}}} \times {\text{small sample correction}}.$$

Using standard error instead of standard deviation in the denominator causes Hedges’ *g* to be overestimated. For example, Seitz et al. [4] calculated Hedges’ *g* values for Wong et al. [19] using standard errors rather than standard deviations, resulting in the outlier described above. For squats, they calculated:

$$g = \frac{{148 {\text{kg}} - 123 {\text{kg}}}}{{1.7{\text{ kg}}}} \times 0.96 = 14.1.$$

Correctly using standard deviation rather than standard error reveals that the effect size for squats is in fact 3.1, not 14.1:

$$g = \frac{{148{\text{ kg}} - 123{\text{ kg}}}}{{7.7 {\text{kg}}}} x \times 0.96 = 3.1.$$

Seitz et al. [4] made the same error when calculating effect sizes from two other papers [15, 16]. These inflated effect sizes led the correlation coefficient and pooled estimates in Seitz et al. [4] to be over-estimated as shown later in the re-analysis.

These errors often result in implausibly large effect sizes and overly narrow effect size confidence intervals (CIs), which are highly conspicuous when graphed, such as in a forest plot (a visual display of the effect sizes and CIs from the underlying studies). Note that in cases where the underlying papers only report the standard errors of the mean, meta-analysts can easily derive the standard deviation — by multiplying the standard error of the mean by the square root of *n*.

### Ignoring Within-Study Correlation

When performing a meta-analysis or meta-regression, some studies may contribute more than one effect size. This can occur because a study includes multiple intervention groups and/or multiple measurements per group. A common error in meta-analyses and meta-regression is to ignore the correlated nature of these observations, which can lead to overly narrow CIs and underestimated *p*-values.

This error had a large impact in Seitz et al. [4]. Seitz et al. [4] included data from just 15 studies in their meta-analysis, but reported 85 effect sizes, and analyzed these effect sizes as if they were completely independent (e.g., calculating a Pearson’s correlation coefficient on the 85 datapoints). The 85 effect sizes arose because some studies included more than one group (e.g., multiple intervention groups and/or a control group) and most studies reported multiple sprint measures per person, for example, reporting the 5-m, 10-m, and 30-m times from a single sprint trial. These sprint measurements are highly correlated; when we re-examined the data, we found that the intra-class correlation coefficient for these measurements was 0.96.

Treating correlated observations as if they are independent can lead one to underestimate standard error, resulting in artificially small *p*-values and artificially narrow CIs. For example, if a study reports six sprint measures per person and these measures are almost perfectly correlated, then treating these six measures as independent effectively inflates the sample size by six-fold, thus leading to vastly underestimated standard errors. Consider also that a single study [17] contributed 36 of the 85 observations in Seitz et al.’s [4] meta-analysis. A single study was therefore treated as if it represented 36 independent studies. Correlated sprint observations are visually apparent in Fig. 1 because they form horizontal stripes of data; we have highlighted three examples with red dashed circles. These horizontal stripes arise when the same group has a single squat effect size (Y value) but multiple, closely spaced sprint effect sizes (X values).

Meta-analysts can account for correlated observations by using an appropriate statistical model, such as a multilevel model. Multilevel meta-analyses account for multiple effect sizes within a study or more generally: when effects within a cluster are more similar to each other than the effect sizes across clusters. When multiple effect sizes are too highly correlated, it may be preferable to select only a single effect size for inclusion. In the case of Seitz et al. [4], we re-analyzed the data using a multilevel model with groups nested within a study to account for the multiple groups per study, but we included only a single sprint measure per group because of the extremely high within-person correlation in sprint times (intra-class correlation coefficient = 0.96).

### Failing to Account for Within-Study Variance

In a meta-analysis or meta-regression, studies are weighted by the amount of information they provide, such that studies that provide more information are weighted more heavily. This is typically done by weighting studies by the inverse of the within-study variance (or within-group variance when there are multiple groups per study). Failure to incorporate this information means that studies will be treated equally regardless of the size of the study.

Seitz et al. [4] do not incorporate information on within-study variance in either their meta-analysis or meta-regression. They appear to instead have run simple linear regression models for all their analyses. Because most studies included in Seitz et al. [4] were similarly small, there was not a huge variation in study weights in this example and thus the impact on results may not have been large. However, this could meaningfully impact in many meta-analyses when sample sizes are more divergent. Researchers attempting to pool effect sizes or perform meta-regression should pick appropriate statistical models that incorporate study weights.

### Focusing on Within-Group Rather Than Between-Group Results

Many meta-analyses include controlled studies but focus more on within-group changes rather than between-group comparisons. This can lead to overly stated results. By comparing to a control group, this removes effects that may have occurred regardless of the intervention (such as placebo effects). Statistically, it is also easier to find significant results using a within-group comparison versus a corresponding between-group comparison [24].

For example, Seitz. et al. [4] include controlled studies in their meta-analysis but only report within-group rather than between-group effect sizes. For example, they report an overall 0.87 standard deviation improvement in sprint performance in groups that received an intervention, but they do not report between-group effect sizes that directly compare the improvements in the intervention groups to their respective control groups. When compared to control groups, the effect size may be smaller. For example, consider the under 15 years of age group in Sander et al. [17]: the sprint effect size for the intervention group was − 1.38, which is large; however, the sprint effect size for the control group was also large: − 0.79. Thus, when the two groups are directly compared, the effect size is only moderate: − 0.5. Meta-analysts should prioritize studies with control groups and should focus on between-group comparisons rather than within-group comparisons.

### Re-Analysis

We re-extracted data from the 15 studies included in Seitz et al. [4]. Data extraction was performed by two independent investigators (DK and KS). We had to exclude the dataset from Tsimahidis et al. [18] as the original study and the requested raw data from the author only provide the percentage change in squat performance and the data presenting the change in kilograms are unavailable. We also made the following additional changes based on the data available in the underlying studies: we (1) added two intervention groups to Rønnestad et al. [16] and deleted the control group datapoint as no control group was found in the original study and (2) added one intervention group and one control group, respectively, to Rønnestad et al. [15]. See the Electronic Supplementary Material (ESM) for the extracted data.

Because of the high correlation between sprint measures from the same group (intra-class correlation coefficient = 0.96), we only included the longest measured sprint distance per group. This left us with 33 effect sizes from 33 groups (24 experimental, 9 control) across 14 studies (as Tsimahidis et al. [18] was excluded). We analyzed the data using a multi-level random-effects model with groups nested within studies using the {metaphor} package in R. (see the ESM for more details and R code).

Seitz et al. [4] reports Hedges’ g effect sizes for the within-group changes in squatting strength and sprint time. They report a correlation coefficient of − 0.773 (95% CI − 0.847, − 0.670) between squat effect size and sprint effect size. In our re-analysis, we found a more moderate correlation of − 0.56, with a much wider 95% CI of − 0.75, − 0.26. Figure 2 shows a plot corresponding to our analysis. We note that there are still several datapoints that have surprisingly large effect sizes (improvements in squat effect size of more than 3 standard deviations). Though we were able to verify that these are the correct values as calculated from the means and standard deviations of the underlying papers, we cannot rule out the presence of errors in the underlying data; for example, some papers report standard deviations that are unexpectedly small for the given measurements.

Though the re-analysis does not change the overall conclusions of the meta-analysis, it does moderate those conclusions — a correlation of 0.773 represents a large correlation in which the majority (59.7%) of variance in sprint improvements can be attributed to increases in lower-body muscle strength whereas 0.56 implies a more moderate correlation in which only a minority (31%) of the variance in sprint improvement can be attributed to increases in lower-body muscle strength. The drop in magnitude is primarily owing to the removal of the Wong et al. [19] outlier. Additionally, importantly, the CI is much less precise. The width of the CI was almost tripled, from 0.18 to 0.49, which is primarily due to the proper accounting for correlated observations and the correct application of a random-effects meta-regression accounting for the between-study heterogeneity.

## Part 2: Frequency of These Errors in Other Highly Cited Meta-Analyses in Strength and Conditioning

To determine how common these errors are in other highly cited meta-analyses, we systematically reviewed 20 of the most highly cited meta-analyses in the field of strength and conditioning research from the past 20 years. We chose strength and conditioning research as previously published MAs utilised incorrect statistical approaches leading to flawed conclusions and practical recommendations [25].

**Fig. 3:**
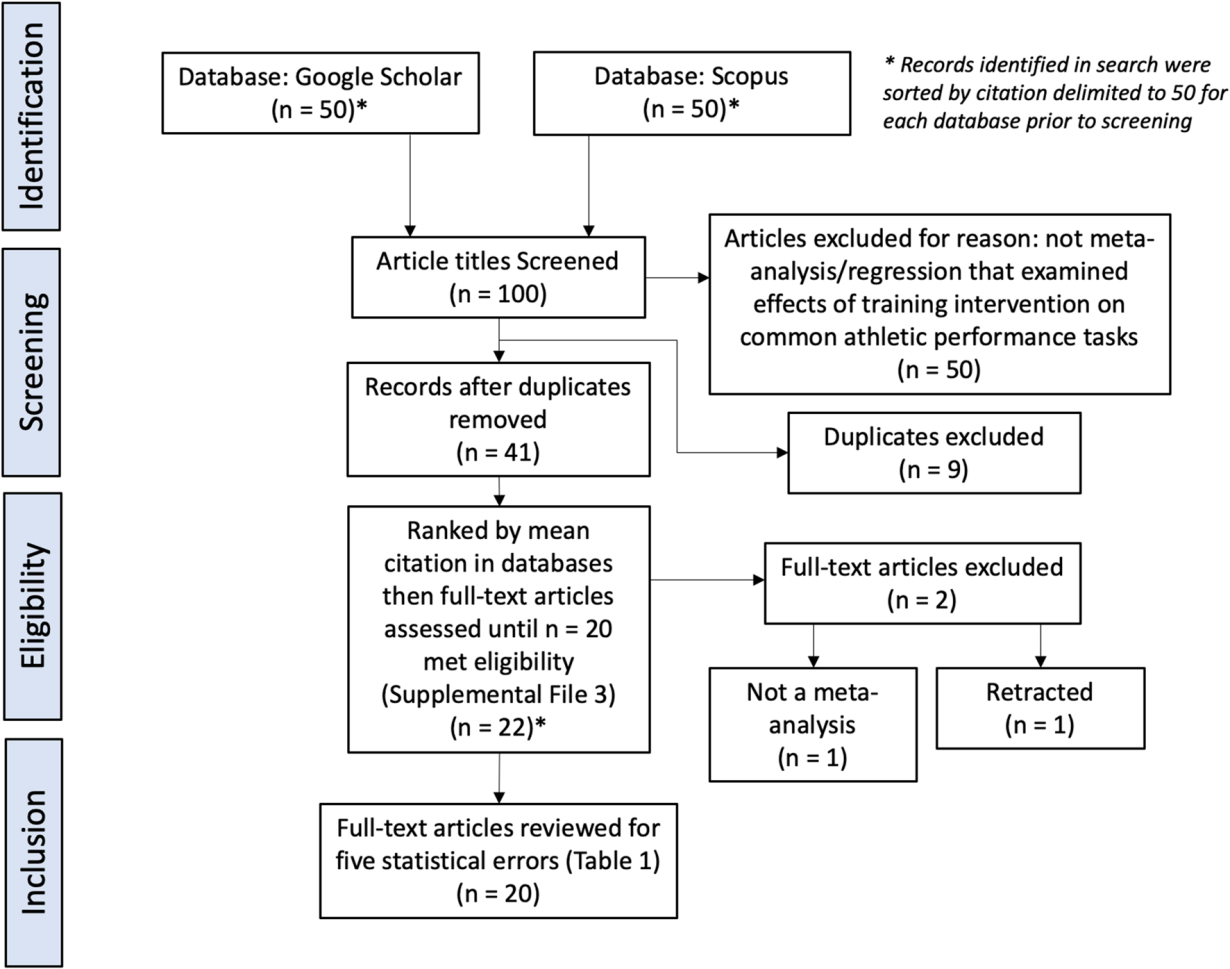
Flow diagram of article inclusion

Our inclusion criteria required a meta-analysis or meta-regression that examined the effects of the training interventions on common athletic performance tasks (e.g., sprint, jump, and throw). Two authors (DK and SN) searched two electronic databases, one highly ranked crawler or easy to use search engine (Google Scholar) and one bibliographic database (SCOPUS) that has a higher capability of repeating search results [26]. The purpose of the search was not a systematic review but merely to identify influential papers by citation (Scopus or Google Scholar citation) over the past 20 years (2000–2020) that would likely have impacted current practice in strength and conditioning. As this article was initially conceived as a teaching article, we did not preregister the methodological approach. The following search terms were used on 19 February, 2021 to identify potential articles: *meta-analysis* OR *meta-regression* AND *strength* OR *resistance* AND *training* AND *athletic* AND *performance* OR *sprint* OR *acceleration* OR *jump* OR *throw*. The search strategy, search results, and excluded articles are summarized in Fig. 3 and provided in the ESM.

We identified the top 20 cited papers (in Google Scholar or Scopus based on mean citations) and systematically reviewed them for the five errors identified in Part 1. Seitz et al. [4] is the ninth most highly cited meta-analysis and contained all five statistical or methodological errors and therefore used as the example in part 1 (Table 1). We defined outliers in the context of this article as standardized effect sizes of greater than 3.0 because an improvement of 3 standard deviations is an implausible effect size for most interventions in strength and conditioning research. Note that the presence of an outlier does not necessarily represent an error — it is the failure to further explore the validity of the datapoint and its impact on results that is the error. All authors (DK, SN, and KS) examined tables, text, and figures to identify such outliers. Five papers [27–31] only reported pooled effect sizes or summary statistics about effect sizes (e.g., means and standard deviations); for these papers, we were unable to evaluate the presence of outliers as we did not have access to the individual effect sizes used in the meta-analyses. For standard error/standard deviation substitutions, it was not possible to check every reported effect size given the large number of effect sizes reported across all 20 studies. Instead, two authors (DK and KS) checked all effect sizes deemed as outliers plus the largest effect sizes from those meta-analyses without outliers by extracting data from the underlying papers. For Williams et al. [32], all effect sizes were graphed in their Fig. 2 but were not linked to specific studies, thus we pulled data from all underlying papers to identify and check the largest effect sizes. We were unable to check for standard deviation/standard error substitutions in the five papers that failed to report individual effect sizes [27–31]. For the remaining three errors, two authors (DK and KS) assessed the statistical approach to determine how correlated observations were handled, what modeling approaches were used, and whether effect sizes reflected within-group or between-group comparisons. Our initial agreement was 93%. Any initial disagreement (see the ESM) between reviewers was resolved by consensus.

**Table 1:** Error identification checklist

#	Title	Year	Mean citation	Outliers (ES > 3.0)	SE/SD error	Failure to account for correlated observations	Failure to weight studies	Focus on within-group rather than between-group comparisons
1	The effects of eccentric versus concentric resistance training on muscle strength and mass in healthy adults: a systematic review with meta-analysis [33]	2009	406	N	N	N	N	N
2	Concurrent training: a meta-analysis examining interference of aerobic and resistance exercises [27]	2012	340	?	?	Y	Y	Y
3	Maximizing strength development in athletes: a meta-analysis to determine the dose–response relationship [28]	2004	282	?	?	Y	Y	Y
4	Does plyometric training improve strength performance? A meta-analysis [34]	2010	275	N	Y	Y	Y	Y
5	Determining variables of plyometric training for improving vertical jump height performance: a meta-analysis [35]	2009	267	N	Y	Y	Y	Y
6	Effects of low-volume high-intensity interval training (HIT) on fitness in adults: a meta-analysis of controlled and non-controlled trials [36]	2014	246	N	N	N	N	N
7	Factors modulating post-activation potentiation of jump, sprint, throw, and upper-body ballistic performances: a systematic review with meta-analysis [29]	2016	156	?	?	Y	Y	Y
8	The effects of plyometric training on sprint performance: a meta-analysis [37]	2012	149	N	Y	Y	Y	Y
9	Increases in lower-body strength transfer positively to sprint performance: a systematic review with meta-analysis [4]	2014	146	Y	Y	Y	Y	Y
10	Systematic review and meta-analysis of linear and undulating periodized resistance training programs on muscular strength [38]	2015	90	N	N	N	N	N
11	Effect of resistance training frequency on gains in muscular strength: a systematic review and meta-analysis [30]	2018	77	?	?	?	N	Y
12	Comparison of periodized and non-periodized resistance training on maximal strength: a meta-analysis [32]	2017	72	Y	N	N	N	N
13	Effect of plyometric training on vertical jump performance in female athletes: a systematic review and meta-analysis [39]	2017	71	Y	Y	N	N	N
14	The effects of rest intervals on jumping performance: a meta-analysis on post-activation potentiation studies [40]	2013	65	N	N	Y	N	N
15	The effects of plyometric training on change-of-direction ability: a meta-analysis [41]	2016	63	N	Y	N	Y	Y
16	The optimal load for maximal power production during lower-body resistance exercises: a meta-analysis [42]	2015	60	Y	Y	N	N	N
17	Olympic weightlifting training improves vertical jump height in sportspeople: a systematic review with meta-analysis [43]	2016	39	N	N	N	N	N
18	The role of trunk muscle strength for physical fitness and athletic performance in trained individuals: a systematic review and meta-analysis [44]	2013	38	Y	Y	N	N	N
19	Strength training for middle-and long-distance performance: a meta-analysis [31]	2018	36	?	?	N	N	N
20	The effectiveness of resisted sled training (RST) for sprint performance: a systematic review and meta-analysis[45]	2018	30	N	Y	Y	N	N
	Error identification summary			5/20 (25%)	9/20 (45%)	9/20 (45%)	8/20 (40%)	9/20 (45%)

*ES* effect size, *N* no error immediately evident, *SD* standard deviation, *SE* standard error, *Y* error immediately evident, *?* unable to evaluate or unclear if error present

As described in the search method, Scopus and Google Scholar were searched for 20 highly cited meta-analysis or meta-regression studies between 2000 and 2020 as of February 2021. All papers identified in the top 20 except Soria-Gila et al. [46] were present in the Scopus and Google Scholar search. The means of Google Scholar and Scopus citations are presented for all papers except Soria-Gila et al. [46]

Table 1 depicts the findings of the systematic review. We excluded one meta-analysis [46] after determining that it was retracted in 2018 [47] because of statistical errors resulting in an incorrect conclusion [48]. We replaced this retracted meta-analysis with the 21st most cited meta-analysis from our search.

**Fig. 4:**
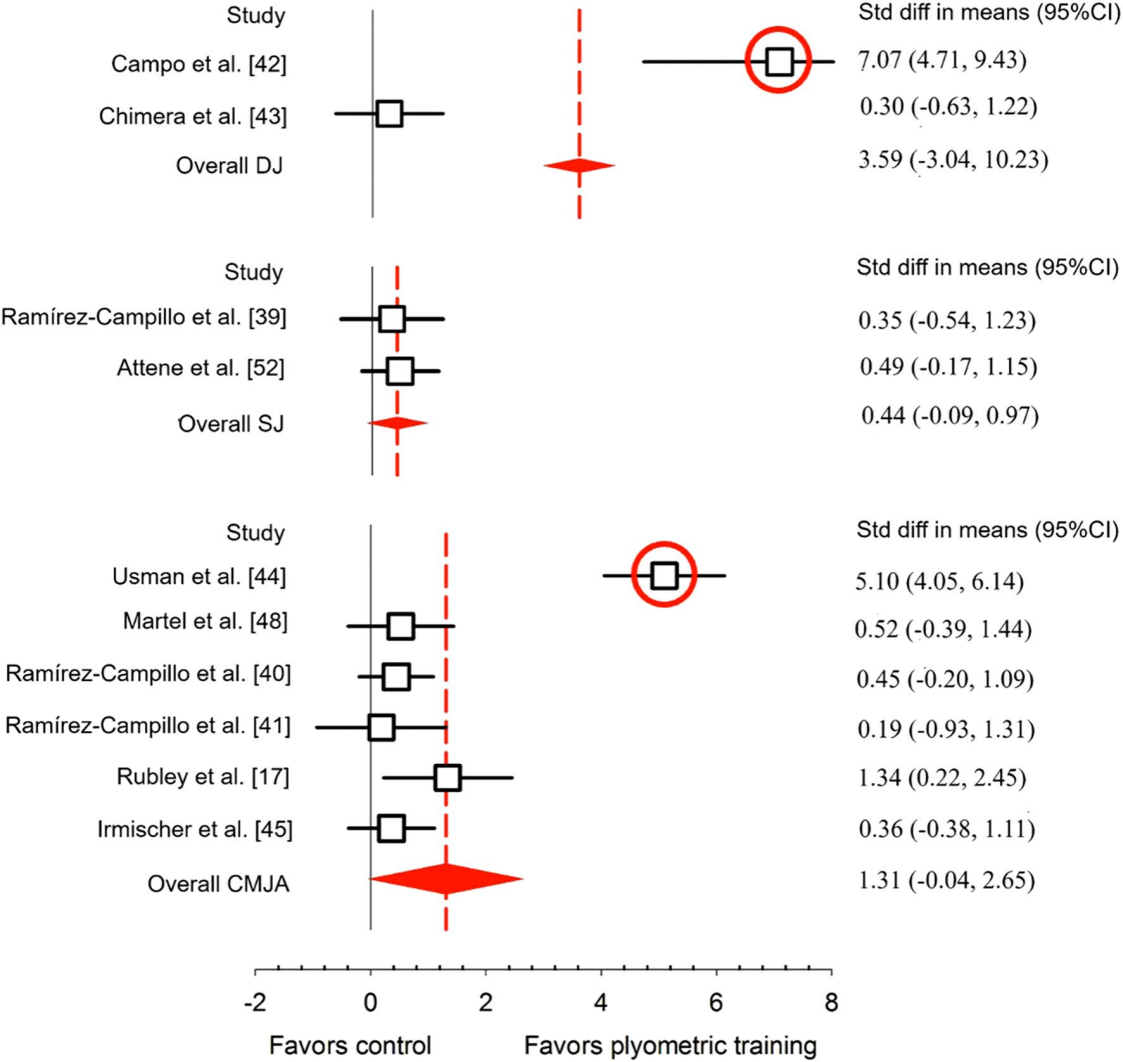
Example forest plot with obvious outliers. “Std difference in means” is short for “standardized difference in means”. *CI* confidence interval, *CMJA* countermovement jump with arm swing, *DJ* drop jump, *SJ* squat jump. From Stojanovic et al. [39]

**Fig. 5:**
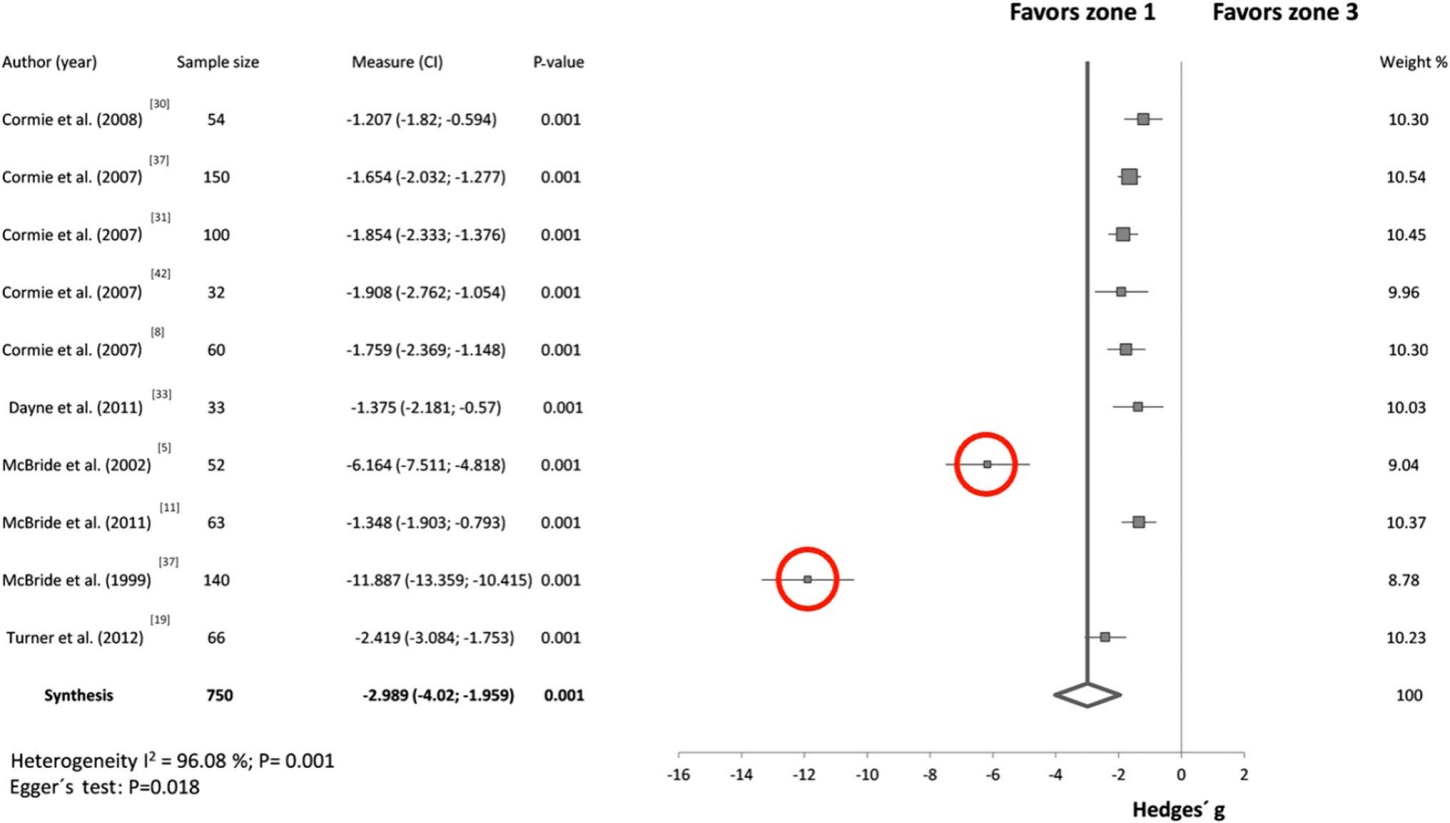
Example forest plot with obvious outliers. *CI* confidence interval. From Soriano et al. [42]

**Fig. 6:**
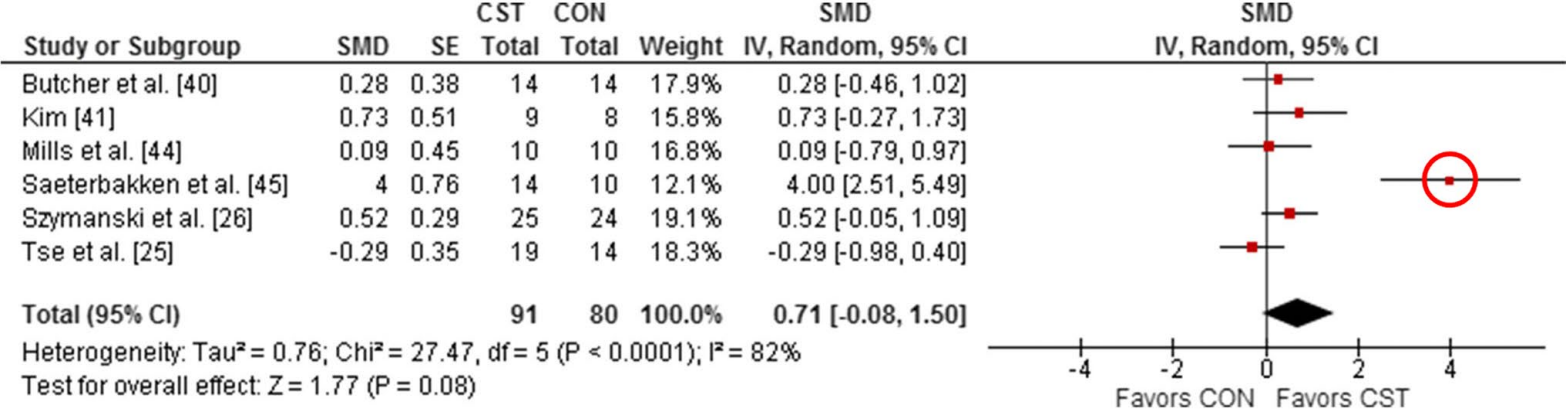
Example forest plot with an obvious outlier. *CI* confidence interval, *CON* control group, *CST* core strength training, *SE* standard error, *SMD* standardized mean difference. From Prieske et al. [44]

In summary, we identified five meta-analyses (25%) with outliers, defined as effect sizes greater than 3.0. For an additional five meta-analyses, we could not determine whether outliers were present as the papers did not report individual effect sizes, but only reported pooled effects or summary statistics about effect sizes (e.g., means and standard deviations of effect sizes across multiple studies). The five meta-analyses with confirmed outliers contained a total of 22 effect sizes greater than 3.0. Of these, 13 (59%) were due to authors miscalculating the effect size using standard error rather than standard deviation (ESM). The explanation for the remaining nine outliers is unclear but we note that some studies had surprisingly low standard deviations and other studies had large effect sizes that are still plausible such as due to maturation in a youth cohort. Figures 4, 5 and 6 show forest plots from three of the papers in our review that contain outliers; these outliers are extremely easy to spot on the forest plots and they all arise from authors accidentally using standard error rather than standard deviation in their calculations.

Nine of 20 meta-analyses (45%) accidentally used standard error rather than standard deviation in the calculation of at least one reported effect size. For an additional five meta-analyses, we could not evaluate whether this error was made because the papers did not report individual effect sizes (as previously described). The ESM shows all effect sizes that were identified to have a standard deviation/standard error as well as their corrected values. We note that in several cases this error resulted in effect sizes that would be considered large but not implausibly large (e.g., effect sizes of about 1.0); these cases are harder to detect. Nine meta-analyses (45%) ignored within-study correlations in their analyses, and we were unable to accurately evaluate whether this error was present in one additional meta-analysis because of a lack of detail in the statistical methods section [30]. Eight meta-analyses (40%) failed to use appropriate meta-analysis or meta-regression techniques to weight studies by the amount of information they contributed. Finally, nine meta-analyses (45%) focused on within-group rather than between-group results.

Though not an error that we systematically searched for, we also identified two meta-analyses [43, 31] that used the standard deviation of the change scores instead of the pooled standard deviation from the pre-testing and post-testing standard deviation to calculate the standardized mean difference. This is problematic because dividing by the standard deviation of the change scores yields information about statistical significance but not about the magnitude of the effect [21]; yet the authors of these meta-analyses incorrectly interpreted the pooled effect sizes as giving information about magnitude. Had the correct standard deviation been used, this likely would have resulted in lower effect sizes.

## Conclusions

Errors in a meta-analysis and meta-regression can substantially impact the calculated results and lead to flawed conclusions. We presented an example meta-regression (Seitz et al. [4]) and highlighted five errors that led to an overestimate of the relationship between increases in squat strength and improvements in sprint performance.

We then systematically reviewed the 20 most highly cited meta-analyses and meta-regression from strength and conditioning research from the past 20 years to assess the frequency of these specific errors. Though these five errors are not an exhaustive list of all possible statistical errors in meta-analyses, they represent errors that are “easy to spot” and often highly impactful. We found that these errors are surprisingly common: of the top 20 most highly cited meta-analyses/meta-regressions in strength and conditioning over the past 20 years, 75% contained at least one of these five statistical errors. An additional 2 out of 20 (another 10%) contained a separate error in the calculation of standardized mean differences (using standard deviation of the change scores). In other words, we identified statistical errors in 85% of the 20 most highly cited meta-analyses in strength and conditioning research over the past 20 years.

Nearly half (45%) of the meta-analyses contained at least one effect size that was overestimated because of the mistaken use of standard error rather than standard deviation in the calculation of effect sizes. This is likely an underestimate of the frequency of this error as (1) we were unable to evaluate this error in 5 of the 20 studies and (2) we did not check every effect size reported from the papers that did report individual effect sizes. In numerous cases, this error resulted in implausibly large and conspicuous effect sizes that arguably should have been caught during peer review (see Fig. 1 and Figs. 4, 5 and 6 for examples). We note that about 60% of all effect sizes > 3.0 were due to a standard error/standard deviation mix-up, meaning that effect sizes > 3.0 should have a high index of suspicion for error. Standard error/standard deviation mix-ups can also result in effect sizes that are large but not implausibly large, for example, effect sizes around 1.0, which may be harder to detect.

Nearly half (45%) of the meta-analyses failed to properly account for correlated observations though many studies included numerous effect sizes from the same study and often from the same group within the same study. For example, Seitz et al. [4] included 85 different effect sizes from just 15 studies, including 36 effect sizes from a single study. This error can cause *p*-values and CI widths to be vastly underestimated.

Forty percent of studies combined effect sizes using simple statistics (e.g., unweighted means) rather than proper techniques for a meta-analysis, which could result in errors due to small studies being given equal weight as large studies. Finally, 45% of studies focused on within-group effects when between-group effects would have been more appropriate, likely leading to overly optimistic results.

## Future Recommendations

1. Understanding common sources of error in meta-analyses helps the reader evaluate published research. We provided an overview of five errors in meta-analyses that can impact the results and conclusions. As such, we first recommend observing the presented data and results (e.g., tables and forest plots) critically for potential outliers. In particular, effect sizes ≥ 3.0 should have a high index of suspicion, as we found that the majority of effect sizes this large arise because of confusing standard error for standard deviation. Assessing the statistical approach can reveal further statistical concerns. In particular, papers should be checked to ensure that they have used appropriate models for meta-analysis/meta-regression and have accounted for correlated observations when applicable. We recommend being particularly critical when the title or the findings are almost “too good to be true” and checking the plausibility of the conclusion based on the presented results and the methodological approach.
2. Providing more transparency when analyzing and interpreting meta-analyses can help minimize errors and flawed conclusions. As such, publicly sharing the procedure to acquire the data itself (e.g., search syntax) and the analytic methods used (e.g., R script) enables others to identify and report potential errors and correct the published conclusion. Further, we recommend that the authors provide all relevant descriptive results with adequate labeling (e.g., mean ± standard deviation) and the de-identified raw data (e.g., as supplementary files) to simplify data extraction for meta-analyses. This procedure ensures and facilitates a more robust and sustainable acquisition and spreading of research outcomes. Similarly, we recommend pre-registering meta-analyses (e.g., Open Science Framework*)* to improve transparency and the confidence of reported findings.
3. Last, the number of such flawed meta-analyses raises serious concerns about the quality of the peer-review process, highlighting a greater need for methodological and statistical expertise when assessing submissions. For meta-analyses, when possible, we recommend collaborating with a statistician to ensure an adequate methodological approach.

## Supplementary Information

Below is the link to the electronic supplementary material.

Supplementary file1 (XLSX 13 kb)

Supplementary file2 (DOCX 38 kb)

Supplementary file3 (DOCX 30 kb)

Supplementary file4 (DOCX 20 kb)

Supplementary file5 (DOCX 24 kb)

## Declarations

### Availability of data and material

The datasets generated and/or analyzed during the current article are openly available in the supplemental files.

### Code availability

See supplemental file 2.
