# Supplementary material for: With Great Power Comes Great Responsibility: Common Errors in Meta-Analyses and Meta-Regressions in Strength & Conditioning Research
Source: Sports Med. 2022 Oct 8;53(2):313–25. doi: 10.1007/s40279-022-01766-0 (PMC9877053; doi:10.1007/s40279-022-01766-0)
Supplement: Supplementary file 2 — Supplementary file2 (DOCX 38 kb) [file 40279_2022_1766_MOESM2_ESM.docx]

**Main findings of the re-analysis:**

Whereas Seitz et al. estimates a correlation between squat ES and sprint ES of -.77, p=.0001, 95% CI: -.847 to -.670, we get r=-0.56, p=.0005, 95% CI: -.75, -.26.

Whereas Seitz. et al. estimate a sprint ES of -0.87 for experimental and 0.02 for control, p<.001 for the difference, we get -0.54 for experimental and -0.01 for control, p=0.007 for the difference.

**Detailed findings and code:**

For the meta-analysis, we fit a multi-level random effects model with groups nested within studies using the metafor package in R.

1. Overall sprint effect size across 14 studies/33 groups: -0.41, p=0.0001, 95% CI: (-0.61, -0.22)


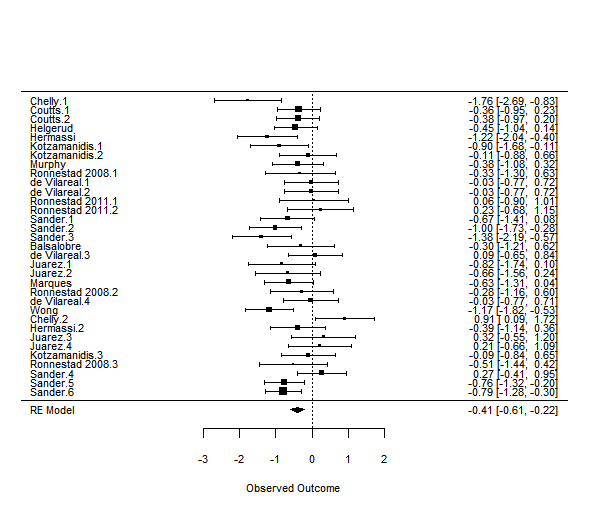


R Code:

#load packages

library(metafor)

library(tidyverse)

library(scales)

library(readxl)

#read data and prepare

data2 <- read_excel('./Supplemental1.xlsx')

head(data2)

labels <- data2$labels

#overall model and forest plot

fit1 <- rma.mv(sprintg,

sprintvarg,

random = ~ 1 |Study/group,

tdist = TRUE,

data = data2,

method = "REML")

summary(fit1)

forest(fit1, slab=labels)

R Output:

Multivariate Meta-Analysis Model (k = 33; method: REML)

logLik Deviance AIC BIC AICc

-25.8109 51.6217 57.6217 62.0189 58.4789

Variance Components:

estim sqrt nlvls fixed factor

sigma^2.1 0.0109 0.1044 14 no Study

sigma^2.2 0.1134 0.3367 33 no Study/group

Test for Heterogeneity:

Q(df = 32) = 60.1415, p-val = 0.0019

Model Results:

estimate se tval pval ci.lb ci.ub

-0.4119 0.0953 -4.3224 0.0001 -0.6061 -0.2178

1. Estimates of sprint ES from experimental (n=24) and control groups (n=9).

Control: ES= -0.01, p= 0.95, 95% CI= (-0.37, 0.35)

Experimental ES: -0.54, p=<.0001, 95% CI= (-0.78, -0.30)

P-value for the difference between groups: .0069

R Code:

#model including experimental group as a predictor

fit2 <- rma.mv(sprintg,

sprintvarg,

random = ~ 1 |Study/group,

tdist = TRUE,

data = data2,

method = "REML", mods=isexp)

summary(fit2)

forest(fit2, slab=labels)

R Output:

Multivariate Meta-Analysis Model (k = 33; method: REML)

logLik Deviance AIC BIC AICc

-22.5678 45.1355 53.1355 58.8715 54.6740

Variance Components:

estim sqrt nlvls fixed factor

sigma^2.1 0.0743 0.2725 14 no Study

sigma^2.2 0.0396 0.1990 33 no Study/group

Test for Residual Heterogeneity:

QE(df = 31) = 56.6096, p-val = 0.0033

Test of Moderators (coefficient 2):

F(df1 = 1, df2 = 31) = 8.3912, p-val = 0.0069

Model Results:

estimate se tval pval ci.lb ci.ub

intrcpt -0.0104 0.1766 -0.0590 0.9534 -0.3706 0.3497

mods -0.5313 0.1834 -2.8968 0.0069 -0.9054 -0.1572 *

1. Estimate of correlation between squat and sprint effect sizes.

r=-0.56, p=.0005, 95% CI: -.26, -.75


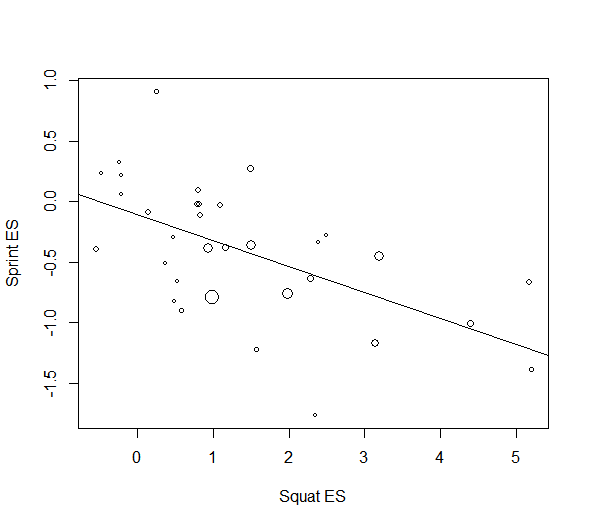


C1. Using multi-level random effects model:

R-squared (from residual heterogeneity) = (60.1415-41.3096)/60.1415=0.313

R=sqrt(.313)=-0.56

p-value = .0005

R code:

fit3 <-rma.mv(sprintg,

sprintvarg,

random = ~ 1 |Study/group,

tdist = TRUE,

data = data2,

method = "REML", mods=squatg)

summary(fit3)

size=rescale(1/data2$sprintvarg,c(0.5,2))

plot(sprintg~squatg,data=data2,cex=size, xlab="Squat ES",

ylab="Sprint ES")

abline(lm(data2$sprintg ~ data2$squatg))

corr=-sqrt((fit1$QE-fit3$QE)/fit1$QE)

print(corr)

R output:

Multivariate Meta-Analysis Model (k = 33; method: REML)

logLik Deviance AIC BIC AICc

-19.2430 38.4859 46.4859 52.2219 48.0244

Variance Components:

estim sqrt nlvls fixed factor

sigma^2.1 0.0000 0.0000 14 no Study

sigma^2.2 0.0443 0.2104 33 no Study/group

Test for Residual Heterogeneity:

QE(df = 31) = 41.3096, p-val = 0.1021

Test of Moderators (coefficient 2):

F(df1 = 1, df2 = 31) = 15.0395, p-val = 0.0005

Model Results:

estimate se tval pval ci.lb ci.ub

intrcpt -0.1213 0.1089 -1.1142 0.2737 -0.3434 0.1007

mods -0.2043 0.0527 -3.8781 0.0005 -0.3118 -0.0969

[1] -0.5595766

C2. Using bivariate random effects model (technically this is more correct because it incorporates the within-study variance for both squat ES and sprint ES, not just sprint ES, but it depends on our estimate of within-study correlation). We get in the same ballpark as above, giving us reassurance that our estimate is reasonable:

Assuming within-study correlation of 0.2: -.45

Assuming within-study correlation of 0.5: -.51

Assuming within-study correlation of 0.8: -.57

R code:

library(metaSEM)

#Add covariances assuming three different within-study correlations

data2$cov1=0.2*sqrt(data2$sprintvarg)*sqrt(data2$squatvarg)

data2$cov2=0.5*sqrt(data2$sprintvarg)*sqrt(data2$squatvarg)

data2$cov3=0.8*sqrt(data2$sprintvarg)*sqrt(data2$squatvarg)

#Run bivariate random effects models assuming different covariance structures

fita <- meta(y=cbind(sprintg, squatg),

v=cbind(sprintvarg, cov1, squatvarg),

data=data2,

model.name="Multi MA")

summary(fita)

VarCorr(fita)

fitb <- meta(y=cbind(sprintg, squatg),

v=cbind(sprintvarg, cov2, squatvarg),

data=data2,

model.name="Multi MA")

summary(fitb)

VarCorr(fitb)

fitc <- meta(y=cbind(sprintg, squatg),

v=cbind(sprintvarg, cov3, squatvarg),

data=data2,

model.name="Multi MA")

summary(fitc)

VarCorr(fitc)

R output:

Number of studies (or clusters): 33

Number of observed statistics: 66

Number of estimated parameters: 5

Degrees of freedom: 61

-2 log likelihood: 155.2282

OpenMx status1: 0 ("0" or "1": The optimization is considered fine.

Other values may indicate problems.)

> VarCorr(fita)

[,1] [,2]

[1,] 0.1341394 -0.4491071

[2,] -0.4491071 1.6021

Number of studies (or clusters): 33

Number of observed statistics: 66

Number of estimated parameters: 5

Degrees of freedom: 61

-2 log likelihood: 155.6512

OpenMx status1: 0 ("0" or "1": The optimization is considered fine.

Other values may indicate problems.)

> VarCorr(fitb)

[,1] [,2]

[1,] 0.1485469 -0.5130042

[2,] -0.5130042 1.6311512

Number of studies (or clusters): 33

Number of observed statistics: 66

Number of estimated parameters: 5

Degrees of freedom: 61

-2 log likelihood: 156.2731

OpenMx status1: 0 ("0" or "1": The optimization is considered fine.

Other values may indicate problems.)

> VarCorr(fitc)

[,1] [,2]

[1,] 0.1641595 -0.5748028

[2,] -0.5748028 1.6715426
