# Supplementary material for: With Great Power Comes Great Responsibility: Common Errors in Meta-Analyses and Meta-Regressions in Strength & Conditioning Research
Source: Sports Med. 2022 Oct 8;53(2):313–25. doi: 10.1007/s40279-022-01766-0 (PMC9877053; doi:10.1007/s40279-022-01766-0)
Supplement: Supplementary file 3 — Supplementary file3 (DOCX 30 kb) [file 40279_2022_1766_MOESM3_ESM.docx]

| **Autor** | **Year** | **Scopus** | **Google Scholar** | **Mean** | **Comment** | **Rank Scopus** | **Rank Google Scholar** | **Rank Mean** |
| --- | --- | --- | --- | --- | --- | --- | --- | --- |
| \| Roig et al. [1] \| 2009 \| 263 \| 548 \| 406 \|  \| 1 \| 1 \| 1 \| \| --- \| --- \| --- \| --- \| --- \| --- \| --- \| --- \| --- \| | 2009 | 263 | 548 | 406 |  | 1 | 1 | 1 |
| Wilson et al. [2] | 2012 | 160 | 519 | 340 |  | 3 | 2 | 2 |
| Peterson et al. [3] | 2004 | 150 | 414 | 282 |  | 4 | 3 | 3 |
| DeVilareal et al. [4] | 2010 |  | 275 | 275 |  | #N/A | 7 | 4 |
| DeVilareal et al. [5] | 2009 | 146 | 388 | 267 |  | 5 | 4 | 5 |
| Weston et al. [6] | 2014 | 181 | 310 | 246 |  | 2 | 5 | 6 |
| Fradkin et al. [7] | 2010 | 132 | 305 | 219 | Excluded as not technically a MA | 6 | 6 | 7 |
| Seitz et al. [8] | 2016 | 99 | 212 | 156 |  | 7 | 8 | 8 |
| DeVilareal et al. [9] | 2012 | 89 | 208 | 149 |  | 9 | 9 | 9 |
| Seitz et al. [10] | 2014 | 92 | 199 | 146 |  | 8 | 10 | 10 |
| Harries et al. [11] | 2015 | 51 | 128 | 90 |  | 10 | 11 | 11 |
| Grgic et al. [12] | 2018 | 50 | 104 | 77 |  | 11 | 13 | 12 |
| Williams et al. [13] | 2017 | 32 | 111 | 72 |  | 16 | 12 | 13 |
| Stojanovic et al. [14] | 2017 | 43 | 98 | 71 |  | 12 | 14 | 14 |
| Gouvea et al. [15] | 2013 | 36 | 94 | 65 |  | 13 | 15 | 15 |
| Asadi et al. [16] | 2016 | 36 | 89 | 63 |  | 13 | 16 | 16 |
| Soriano et al. [17] | 2015 | 36 | 83 | 60 |  | 13 | 17 | 17 |
| Soria-Gila et al. [18] | 2015 | 24 | 54 | 39 | This MA was retracted | 17 | 19 | 18 |
| Hackett et al. [19] | 2016 | 15 | 63 | 39 |  | 21 | 18 | 18 |
| Prieske et al. [20] | 2016 | 21 | 54 | 38 |  | 18 | 19 | 20 |
| Beryman et al. [21] | 2018 | 18 | 53 | 36 | Added due to Fradkin et al [7] exclusion | 20 | 21 | 21 |
| Alcaraz et al. [22] | 2018 | 21 | 39 | 30 | Added due to Soria-Gila et al [18] retraction | 18 | 22 | 22 |

Supplement table 2. Meta-analysis ranking

Supplement table 3. Initial agreement overview (DK and KS) and final decision for each MA

|  | error 1 | | error 2 | | error 3 | | error 4 | | error 5 | |
| --- | --- | --- | --- | --- | --- | --- | --- | --- | --- | --- |
|  | DK | KS | DK | KS | DK | KS | DK | KS | DK | KS |
| Roig et al. [1] | N | | N | | N | | N | | N | |
|  | N | N | N | N | N | N | N | N | N | N |
| Wilson et al. [2] | ? | | ? | | Y | | Y | | Y | |
|  | ? | ? | ? | ? | Y | Y | Y | Y | Y | Y |
| Peterson et al. [3] | ? | | ? | | Y | | Y | | Y | |
|  | ? | ? | ? | ? | Y | Y | Y | Y | Y | Y |
| DeVilareal et al. [4] | N | | Y | | Y | | Y | | Y | |
|  | N | N | Y | N | Y | Y | Y | Y | Y | Y |
| DeVilareal et al. [5] | N | | Y | | Y | | Y | | Y | |
|  | N | N | Y | Y | Y | Y | Y | Y | Y | Y |
| Weston et al. [6] | N | | N | | N | | N | | N | |
|  | N | N | N | N | N | N | N | N | Y | N |
| Seitz et al. [8] | ? | | ? | | Y | | Y | | Y | |
|  | ? | ? | ? | ? | Y | Y | Y | Y | Y | Y |
| DeVilareal et al. [9] | N | | Y | | Y | | Y | | Y | |
|  | N | N | Y | Y | Y | Y | Y | Y | Y | Y |
| Seitz et al. [10] | Y | | Y | | Y | | Y | | Y | |
|  | Y | Y | Y | Y | Y | Y | Y | Y | Y | Y |
| Harries et al. [11] | N | | N | | N | | N | | N | |
|  | N | N | N | N | N | N | N | N | ? | N |
| Grgic et al. [12] | ? | | ? | | ? | | N | | Y | |
|  | ? | ? | ? | ? | Y | ? | N | N | Y | Y |
| Williams et al. [13] | Y | | N | | N | | N | | N | |
|  | Y | Y | ? | N | N | N | N | N | N | N |
| Stojanovic et al. [14] | Y | | Y | | N | | N | | N | |
|  | Y | Y | Y | Y | N | N | N | N | N | N |
| Gouvea et al. [15] | N | | N | | Y | | N | | N | |
|  | N | N | N | N | Y | Y | N | N | N | N |
| Asadi et al. [16] | N | | Y | | N | | Y | | Y | |
|  | N | N | Y | Y | N | N | Y | Y | Y | Y |
| Soriano et al. [17] | Y | | Y | | N | | N | | N | |
|  | Y | Y | Y | Y | N | N | N | N | N | N |
| Hackett et al. [19] | N | | N | | N | | N | | N | |
|  | N | N | N | N | N | N | N | N | N | N |
| Prieske et al. [20] | Y | | Y | | N | | N | | N | |
|  | Y | Y | Y | Y | N | N | N | N | N | N |
| Berryman et al. [21] | ? | | ? | | N | | N | | N | |
|  | ? | N | ? | N | N | N | N | N | N | N |
| Alcaraz et al. [22] | N | | Y | | Y | | N | | N | |
|  | N | N | Y | Y | Y | Y | N | N | N | N |

Note: N = no error evident; Y = error evident; ? = unable to evaluate or unclear if error present. Green shading shows the final agreement, yellow shading shows an initial disagreement, no shading indicates initial agreement.

References:

1. Roig M, O’Brien K, Kirk G, Murray R, McKinnon P, Shadgan B, et al. The effects of eccentric versus concentric resistance training on muscle strength and mass in healthy adults: a systematic review with meta-analysis. *British Journal of Sports Medicine*. 2009;43(8): 556–568. [https://doi.org/DOI 10.1136/bjsm.2008.051417](https://doi.org/DOI%2010.1136/bjsm.2008.051417).

2. Wilson JM, Marin PJ, Rhea MR, Wilson SMC, Loenneke JP, Anderson JC. Concurrent training: a meta-analysis examining interference of aerobic and resistance exercises. *Journal of Strength and Conditioning Research*. 2012;26(8): 2293–2307. <https://doi.org/10.1519/JSC.0b013e31823a3e2d>.

3. Peterson MD, Rhea MR, Alvar BA. Maximizing strength development in athletes: a meta-analysis to determine the dose-response relationship. *Journal of Strength and Conditioning Research*. 2004;18(2): 377–382. <https://doi.org/10.1519/R-12842.1>.

4. de Villarreal ES-S, Requena B, Newton RU. Does plyometric training improve strength performance? A meta-analysis. *Journal of Science and Medicine in Sport*. 2010;13(5): 513–522. <https://doi.org/10.1016/j.jsams.2009.08.005>.

5. de Villarreal ES, Kellis E, Kraemer WJ, Izquierdo M. Determining variables of plyometric training for improving vertical jump height performance: a meta-analysis. *Journal of Strength and Conditioning Research*. 2009;23(2): 495–506. <https://doi.org/10.1519/JSC.0b013e318196b7c6>.

6. Weston M, Taylor KL, Batterham AM, Hopkins WG. Effects of low-volume high-intensity interval training (HIT) on fitness in adults: a meta-analysis of controlled and non-controlled trials. *Sports Medicine*. 2014;44(7): 1005–1017. <https://doi.org/10.1007/s40279-014-0180-z>.

7. Fradkin AJ, Zazryn TR, Smoliga JM. Effects of warming-up on physical performance: a systematic review with meta-analysis. *Journal of Strength and Conditioning Research*. 2010;24(1): 140–148. <https://doi.org/10.1519/JSC.0b013e3181c643a0>.

8. Seitz LB, Haff GG. Factors modulating post-activation potentiation of jump, sprint, throw, and upper-body ballistic performances: a systematic review with meta-analysis. *Sports Medicine*. 2016;46(2): 231–240. <https://doi.org/10.1007/s40279-015-0415-7>.

9. de Villarreal ES, Requena B, Cronin JB. The effects of plyometric training on sprint performance: a meta-analysis. *Journal of Strength and Conditioning Research*. 2012;26(2): 575–584. <https://doi.org/10.1519/JSC.0b013e318220fd03>.

10. Seitz LB, Reyes A, Tran TT, Saez de Villarreal E, Haff GG. Increases in lower-body strength transfer positively to sprint performance: a systematic review with meta-analysis. *Sports Medicine*. 2014;44(12): 1693–1702. <https://doi.org/10.1007/s40279-014-0227-1>.

11. Harries SK, Lubans DR, Callister R. Systematic review and meta-analysis of linear and undulating periodized resistance training programs on muscular strength. *Journal of Strength and Conditioning Research*. 2015;29(4): 1113–1125. <https://doi.org/10.1519/JSC.0000000000000712>.

12. Grgic J, Schoenfeld BJ, Davies TB, Lazinica B, Krieger JW, Pedisic Z. Effect of resistance training frequency on gains in muscular strength: a systematic review and meta-analysis. *Sports Medicine (Auckland, N.Z.)*. 2018;48(5): 1207–1220. <https://doi.org/10.1007/s40279-018-0872-x>.

13. Williams TD, Tolusso DV, Fedewa MV, Esco MR. Comparison of periodized and non-periodized resistance training on maximal strength: a meta-analysis. *Sports Medicine (Auckland, N.Z.)*. 2017;47(10): 2083–2100. <https://doi.org/10.1007/s40279-017-0734-y>.

14. Stojanović E, Ristić V, McMaster DT, Milanović Z. Effect of plyometric training on vertical jump performance in female athletes: a systematic review and meta-analysis. *Sports Medicine (Auckland, N.Z.)*. 2017;47(5): 975–986. <https://doi.org/10.1007/s40279-016-0634-6>.

15. Gouvêa AL, Fernandes IA, César EP, Silva WAB, Gomes PSC. The effects of rest intervals on jumping performance: a meta-analysis on post-activation potentiation studies. *Journal of Sports Sciences*. 2013;31(5): 459–467. <https://doi.org/10.1080/02640414.2012.738924>.

16. Asadi A, Arazi H, Young WB, Sáez de Villarreal E. The effects of plyometric training on change-of-direction ability: a meta-analysis. *International Journal of Sports Physiology and Performance*. 2016;11(5): 563–573. <https://doi.org/10.1123/ijspp.2015-0694>.

17. Soriano MA, Jiménez-Reyes P, Rhea MR, Marín PJ. The optimal load for maximal power production during lower-body resistance exercises: a meta-analysis. *Sports Medicine (Auckland, N.Z.)*. 2015;45(8): 1191–1205. <https://doi.org/10.1007/s40279-015-0341-8>.

18. Soria-Gila MA, Chirosa IJ, Bautista IJ, Baena S, Chirosa LJ. Effects of variable resistance training on maximal strength: a meta-analysis. *Journal of Strength and Conditioning Research*. 2015;29(11): 3260–3270. <https://doi.org/10.1519/JSC.0000000000000971>.

19. Hackett D, Davies T, Soomro N, Halaki M. Olympic weightlifting training improves vertical jump height in sportspeople: a systematic review with meta-analysis. *British Journal of Sports Medicine*. 2016;50(14): 865–872. <https://doi.org/10.1136/bjsports-2015-094951>.

20. Prieske O, Muehlbauer T, Granacher U. The role of trunk muscle strength for physical fitness and athletic performance in trained individuals: a systematic review and meta-analysis. *Sports Medicine (Auckland, N.Z.)*. 2016;46(3): 401–419. <https://doi.org/10.1007/s40279-015-0426-4>.

21. Berryman N, Mujika I, Arvisais D, Roubeix M, Binet C, Bosquet L. Strength training for middle- and long-distance performance: a meta-analysis. *International Journal of Sports Physiology and Performance*. 2018;13(1): 57–63. <https://doi.org/10.1123/ijspp.2017-0032>.

22. Alcaraz PE, Carlos-Vivas J, Oponjuru BO, Martínez-Rodríguez A. The effectiveness of resisted sled training (RST) for sprint performance: a systematic review and meta-analysis. *Sports Medicine (Auckland, N.Z.)*. 2018;48(9): 2143–2165. <https://doi.org/10.1007/s40279-018-0947-8>.
