# Supplementary material for: With Great Power Comes Great Responsibility: Common Errors in Meta-Analyses and Meta-Regressions in Strength & Conditioning Research
Source: Sports Med. 2022 Oct 8;53(2):313–25. doi: 10.1007/s40279-022-01766-0 (PMC9877053; doi:10.1007/s40279-022-01766-0)
Supplement: Supplementary file 4 — Supplementary file4 (DOCX 20 kb) [file 40279_2022_1766_MOESM4_ESM.docx]

Supplement table 4. All effect sizes $\geq3$

| **Meta-Analysis** | **Effect Size/s reported in the MA** | **Underlying Paper** | **SE/SD error?** |
| --- | --- | --- | --- |
| Seitz et al. [1] | 15, -5.3 | Wong et al[6] | YES |
| Seitz et al. [1] | 3.34 | Helgerud et al[7] | NO |
| Seitz et al. [1] | 5.53, 4.71, 5.63 | Sander et al[8] | NO |
| Willliams et al. [2] | 4.8 | Monetrio et al[9] | NO |
| Willliams et al. [2] | 4.6, 3.36 | Marx et al[10] | NO |
| Stojanovic et al. [3] | 3.36 | Ozbar et al[11] | NO |
| Stojanovic et al. [3] | 4.20, 7.07 | Campo et al[12] | YES |
| Stojanovic et al. [3] | 5.1 | Usman et al[13] | YES |
| Soriano et al. [4] | -11.88, -4.18, -8.01 | McBride et al[14] | YES |
| Soriano et al. [4] | -6.16, -4.14, -3.14 | McBride et al[15] | YES |
| Prieske et al. [5] | 3.75 | Durall et al[16] | YES |
| Prieske et al. [5] | 3.36 | Stanton et al[17] | NO |
| Prieske et al. [5] | 4 | Saeterbakken et al[18] | YES |

References

1. Seitz LB, Reyes A, Tran TT, Saez de Villarreal E, Haff GG. Increases in lower-body strength transfer positively to sprint performance: a systematic review with meta-analysis. *Sports Medicine*. 2014;44(12): 1693–1702. <https://doi.org/10.1007/s40279-014-0227-1>.

2. Williams TD, Tolusso DV, Fedewa MV, Esco MR. Comparison of periodized and non-periodized resistance training on maximal strength: a meta-analysis. *Sports Medicine (Auckland, N.Z.)*. 2017;47(10): 2083–2100. <https://doi.org/10.1007/s40279-017-0734-y>.

3. Stojanović E, Ristić V, McMaster DT, Milanović Z. Effect of plyometric training on vertical jump performance in female athletes: a systematic review and meta-analysis. *Sports Medicine (Auckland, N.Z.)*. 2017;47(5): 975–986. <https://doi.org/10.1007/s40279-016-0634-6>.

4. Soriano MA, Jiménez-Reyes P, Rhea MR, Marín PJ. The optimal load for maximal power production during lower-body resistance exercises: a meta-analysis. *Sports Medicine (Auckland, N.Z.)*. 2015;45(8): 1191–1205. <https://doi.org/10.1007/s40279-015-0341-8>.

5. Prieske O, Muehlbauer T, Granacher U. The role of trunk muscle strength for physical fitness and athletic performance in trained individuals: a systematic review and meta-analysis. *Sports Medicine (Auckland, N.Z.)*. 2016;46(3): 401–419. <https://doi.org/10.1007/s40279-015-0426-4>.

6. Wong P, Chaouachi A, Chamari K, Dellal A, Wisloff U. Effect of preseason concurrent muscular strength and high-intensity interval training in professional soccer players. *Journal of Strength and Conditioning Research*. 2010;24(3): 653–660. <https://doi.org/10.1519/JSC.0b013e3181aa36a2>.

7. Helgerud J, Rodas G, Kemi OJ, Hoff J. Strength and endurance in elite football players. *International Journal of Sports Medicine*. 2011;32(09): 677–682. <https://doi.org/10.1055/s-0031-1275742>.

8. Sander A, Keiner M, Wirth K, Schmidtbleicher D. Influence of a 2-year strength training programme on power performance in elite youth soccer players. *European Journal of Sport Science*. 2013;13(5): 445–451. <https://doi.org/10.1080/17461391.2012.742572>.

9. Monteiro AG, Aoki MS, Evangelista AL, Alveno DA, Monteiro GA, Piçarro I da C, et al. Nonlinear periodization maximizes strength gains in split resistance training routines. *Journal of Strength and Conditioning Research*. 2009;23(4): 1321–1326. <https://doi.org/10.1519/JSC.0b013e3181a00f96>.

10. Marx JO, Ratamess NA, Nindl BC, Gotshalk LA, Volek JS, Dohi K, et al. Low-volume circuit versus high-volume periodized resistance training in women: *Medicine and Science in Sports and Exercise*. 2001; 635–643. <https://doi.org/10.1097/00005768-200104000-00019>.

11. Ozbar N. Effects of plyometric training on explosive strength, speed and kicking speed in female soccer players. *The Anthropologist*. 2015;19(2): 333–339. <https://doi.org/10.1080/09720073.2015.11891666>.

12. Campo SS, Vaeyens R, Philippaerts RM, Redondo JC, de Benito AM, Cuadrado G. Effects of lower-limb plyometric training on body composition, explosive strength, and kicking speed in female soccer players. *Journal of Strength and Conditioning Research*. 2009;23(6): 1714–1722. <https://doi.org/10.1519/JSC.0b013e3181b3f537>.

13. Usman T, Shenoy K. Effects of lower body plyometric training on vertical jump performance and pulmonary function in male and female collegiate volleyball players. *International Journal of Applied Exercise Physiology*. 2015;4(2):9–19.

14. McBride JM, Triplett-McBride T, Davie A, Newton RU. A comparison of strength and power characteristics between power lifters, olympic lifters, and sprinters. *The Journal of Strength & Conditioning Research*. 1999;13(1): 58–66.

15. McBride JM, Triplett-McBride T, Davie A, Newton RU. The effect of heavy- vs. light-load jump squats on the development of strength, power, and speed. *Journal of Strength and Conditioning Research*. 2002;16(1): 75–82.

16. Durall CJ, Udermann BE, Johansen DR, Gibson B, Reineke DM, Reuteman P. The effects of preseason trunk muscle training on low-back pain occurrence in women collegiate gymnasts. *Journal of Strength and Conditioning Research*. 2009;23(1): 86–92. <https://doi.org/10.1519/JSC.0b013e31818b93ac>.

17. Stanton R, Reaburn PR, Humphries B. The effect of short-term Swiss ball training on core stability and running economy. *Journal of Strength and Conditioning Research*. 2004;18(3): 522–528. <https://doi.org/10.1519/1533-4287>.

18. Saeterbakken AH, van den Tillaar R, Seiler S. Effect of core stability training on throwing velocity in female handball players. *Journal of Strength and Conditioning Research*. 2011;25(3): 712–718. <https://doi.org/10.1519/JSC.0b013e3181cc227e>.
