# Supplementary material for: With Great Power Comes Great Responsibility: Common Errors in Meta-Analyses and Meta-Regressions in Strength & Conditioning Research
Source: Sports Med. 2022 Oct 8;53(2):313–25. doi: 10.1007/s40279-022-01766-0 (PMC9877053; doi:10.1007/s40279-022-01766-0)
Supplement: Supplementary file 5 — Supplementary file5 (DOCX 24 kb) [file 40279_2022_1766_MOESM5_ESM.docx]

Supplement table 5. All SE/SD errors identified

| **Meta-Analysis** | **Underlying Paper** | **Group** | **Task** | **ES with the SE/SD error** | **Corrected ES (Hedge's g)** |  |
| --- | --- | --- | --- | --- | --- | --- |
| DeVilareal et al. [1] | Herrero et al. [10] | EPG | Sprint | 1.69 | 0.72 |  |
| DeVilareal et al. [1] | Herrero et al. [10] | PG | Sprint | 0.13 | 0.05 |  |
| DeVilareal et al. [2] | Fatouros et al. [11] | PT | Countermovement jump | 2.25 | 0.74 |  |
| DeVilareal et al. [2] | Fatouros et al. [11] | PWT | Countermovement jump | 2.57 | 0.89 |  |
| DeVilareal et al. [3] | Ronnestad et al. [12] | Pooled intervention group | Sprint | 0.93 | 0.31 |  |
| Seitz et al. [4] | Wong et al. [13] | EG | Squat | -5.3 | -0.9 |  |
| Seitz et al. [4] | Wong et al. [13] | EG | Sprint | 14.1 | 3.1 |  |
| Seitz et al. [4] | Ronnestadt et al. [12] | STR | Sprint | -1.55 | -0.2 |  |
| Stojanovic et al. [5] | Campo et al. [14] | PG | Countermovement jump | 4.2 | 1.14 |  |
| Stojanovic et al. [5] | Campo et al. [14] | PG | Drop jump | 7.07 | 1.28 |  |
| Stojanovic et al. [5] | Usman et al. [15] | Group III | Countermovement jump | 5.1 | 1 |  |
| Asadi et al. [6] | Malisoux et al. [16] | Experimental | Shuttle run | 2.1 | -0.89 |  |
| Soriano et al. [7] | McBride et al. [17] | Zone 1 and Zone 2 | Jump squat | -8.007 | -3.00* |  |
| Soriano et al. [7] | McBride et al. [17] | Zone 1 and Zone 3 | Jump squat | -11.887 | -4.29* |  |
| Soriano et al. [7] | McBride et al. [17] | Zone 2 and Zone 3 | Jump squat | -4.18 | -2.76* |  |
| Soriano et al. [7] | McBride et al. [18] | Zone 1 and Zone 2 | Jump squat | -3.142 | -0.49* |  |
| Soriano et al. [7] | McBride et al. [18] | Zone 1 and Zone 3 | Jump squat | -6.164 | -1.73* |  |
| Soriano et al. [7] | McBride et al. [18] | Zone 2 and Zone 3 | Jump squat | -4.144 | -1.72* |  |
| Prieske et al. [8] | Durall et al. [19] | Training | Side bridge right | 3.75 | 1.79* |  |
| Prieske et al. [8] | Saeterbakken et al. [21] | SET | Throwing velocity | 4 | 0.52 |  |
| Alcaraz et al. [9] | Zafeiridis et al. [22] | RST - Pre and post test | Sprint | 1.92 | 0.58 |  |
| * impossible to determine what datapoint contributed to the presented ES so we calculated the biggest ES possible from the underlying dataset; | | | | | | |
| SE = Standard error; SD = Standard deviation; ES = Effect size; EPG = Electromyostimulation and plymetric group; PG = Plyometric group; PT = Plyometric training; PWT = Plyometric and weight training; EG = Experimental group; CG = Control group; SET = Swissball experimental training; CON = Control goup; RST = Resisted sprint training | | | | | | |
|  | | | | | | |

References

1. de Villarreal ES-S, Requena B, Newton RU. Does plyometric training improve strength performance? A meta-analysis. *Journal of Science and Medicine in Sport*. 2010;13(5): 513–522. <https://doi.org/10.1016/j.jsams.2009.08.005>.

2. de Villarreal ES, Kellis E, Kraemer WJ, Izquierdo M. Determining variables of plyometric training for improving vertical jump height performance: a meta-analysis. *Journal of Strength and Conditioning Research*. 2009;23(2): 495–506. <https://doi.org/10.1519/JSC.0b013e318196b7c6>.

3. de Villarreal ES, Requena B, Cronin JB. The effects of plyometric training on sprint performance: a meta-analysis. *Journal of Strength and Conditioning Research*. 2012;26(2): 575–584. <https://doi.org/10.1519/JSC.0b013e318220fd03>.

4. Seitz LB, Reyes A, Tran TT, Saez de Villarreal E, Haff GG. Increases in lower-body strength transfer positively to sprint performance: a systematic review with meta-analysis. *Sports Medicine*. 2014;44(12): 1693–1702. <https://doi.org/10.1007/s40279-014-0227-1>.

5. Stojanović E, Ristić V, McMaster DT, Milanović Z. Effect of plyometric training on vertical jump performance in female athletes: a systematic review and meta-analysis. *Sports Medicine (Auckland, N.Z.)*. 2017;47(5): 975–986. <https://doi.org/10.1007/s40279-016-0634-6>.

6. Asadi A, Arazi H, Young WB, Sáez de Villarreal E. The effects of plyometric training on change-of-direction ability: a meta-analysis. *International Journal of Sports Physiology and Performance*. 2016;11(5): 563–573. <https://doi.org/10.1123/ijspp.2015-0694>.

7. Soriano MA, Jiménez-Reyes P, Rhea MR, Marín PJ. The optimal load for maximal power production during lower-body resistance exercises: a meta-analysis. *Sports Medicine (Auckland, N.Z.)*. 2015;45(8): 1191–1205. <https://doi.org/10.1007/s40279-015-0341-8>.

8. Prieske O, Muehlbauer T, Granacher U. The role of trunk muscle strength for physical fitness and athletic performance in trained individuals: a systematic review and meta-analysis. *Sports Medicine (Auckland, N.Z.)*. 2016;46(3): 401–419. <https://doi.org/10.1007/s40279-015-0426-4>.

9. Alcaraz PE, Carlos-Vivas J, Oponjuru BO, Martínez-Rodríguez A. The effectiveness of resisted sled training (RST) for sprint performance: a systematic review and meta-analysis. *Sports Medicine (Auckland, N.Z.)*. 2018;48(9): 2143–2165. <https://doi.org/10.1007/s40279-018-0947-8>.

10. Herrero J, Izquierdo M, Maffiuletti N, García-López J. Electromyostimulation and plyometric training effects on jumping and sprint time. *International Journal of Sports Medicine*. 2006;27(7): 533–539. <https://doi.org/10.1055/s-2005-865845>.

11. Fatouros IG, Jamurtas AZ, Leontsini D, Taxildaris K, Aggelousis N, Kostopoulos N, et al. Evaluation of plyometric exercise training, weight training, and their combination on vertical jumping performance and leg strength. Journal of Strength and Conditioning Research. 2000;14(4): 470-476. https://doi.org/[10.1519/00124278-200011000-00016](http://dx.doi.org/10.1519/00124278-200011000-00016)

12. Rønnestad BR, Kvamme NH, Sunde A, Raastad T. Short-term effects of strength and plyometric training on sprint and jump performance in professional soccer players. *Journal of Strength and Conditioning Research*. 2008;22(3): 773–780. <https://doi.org/10.1519/JSC.0b013e31816a5e86>.

13. Wong P, Chaouachi A, Chamari K, Dellal A, Wisloff U. Effect of preseason concurrent muscular strength and high-intensity interval training in professional soccer players. *Journal of Strength and Conditioning Research*. 2010;24(3): 653–660. <https://doi.org/10.1519/JSC.0b013e3181aa36a2>.

14. Campo SS, Vaeyens R, Philippaerts RM, Redondo JC, de Benito AM, Cuadrado G. Effects of lower-limb plyometric training on body composition, explosive strength, and kicking speed in female soccer players. *Journal of Strength and Conditioning Research*. 2009;23(6): 1714–1722. <https://doi.org/10.1519/JSC.0b013e3181b3f537>.

15. Usman T, Shenoy K. Effects of lower body plyometric training on vertical jump performance and pulmonary function in male and female collegiate volleyball players. *International Journal of Applied Exercise Physiology*. 2015;4(2):9–19.

16. Malisoux L, Francaux M, Nielens H, Theisen D. Stretch-shortening cycle exercises: an effective training paradigm to enhance power output of human single muscle fibers. *Journal of Applied Physiology*. 2006;100(3): 771–779. <https://doi.org/10.1152/japplphysiol.01027.2005>.

17. McBride JM, Triplett-McBride T, Davie A, Newton RU. A comparison of strength and power characteristics between power lifters, olympic lifters, and sprinters. *The Journal of Strength & Conditioning Research*. 1999;13(1): 58–66.

18. McBride JM, Triplett-McBride T, Davie A, Newton RU. The effect of heavy- vs. light-load jump squats on the development of strength, power, and speed. *Journal of Strength and Conditioning Research*. 2002;16(1): 75–82.

19. Durall CJ, Udermann BE, Johansen DR, Gibson B, Reineke DM, Reuteman P. The effects of preseason trunk muscle training on low-back pain occurrence in women collegiate gymnasts. *Journal of Strength and Conditioning Research*. 2009;23(1): 86–92. <https://doi.org/10.1519/JSC.0b013e31818b93ac>.

20. Stanton R, Reaburn PR, Humphries B. The effect of short-term Swiss ball training on core stability and running economy. *Journal of Strength and Conditioning Research*. 2004;18(3): 522–528. <https://doi.org/10.1519/1533-4287>.

21. Saeterbakken AH, van den Tillaar R, Seiler S. Effect of core stability training on throwing velocity in female handball players. *Journal of Strength and Conditioning Research*. 2011;25(3): 712–718. <https://doi.org/10.1519/JSC.0b013e3181cc227e>.

22. Zafeiridis A, Saraslanidis P, Manou V, Ioakimidis P, Dipla K, Kellis S. The effects of resisted sled-pulling sprint training on acceleration and maximum speed performance. *The Journal of Sports Medicine and Physical Fitness*. 2005;45(3): 284–290.
